# Genomic structural equation study reveals links between anorexia nervosa and delay discounting and lack of perseverance but not other facets of impulsivity

**DOI:** 10.3389/fpsyt.2025.1613776

**Published:** 2025-07-04

**Authors:** Sevim B. Bianchi, Laura Vilar-Ribó, Abraham A. Palmer, Daniel E. Gustavson, Sandra Sanchez-Roige

**Affiliations:** ^1^ Department of Psychiatry, University of California San Diego, La Jolla, CA, United States; ^2^ College of Medicine, California Northstate University, Elk Grove, CA, United States; ^3^ Institute for Genomic Medicine, University of California San Diego, La Jolla, CA, United States; ^4^ Institute for Behavioral Genetics, University of Colorado Boulder, Boulder, CO, United States; ^5^ Department of Medicine, Division of Genetic Medicine, Vanderbilt University, Nashville, TN, United States

**Keywords:** genomic structural equation modeling, GWAS, eating disorders, anorexia, delay discounting, BIS, UPPS, impulsivity

## Abstract

Anorexia nervosa (AN) is a heritable condition, characterized by a fear of weight gain and a distorted body image, for which treatments are only limited. AN is characterized by excessive control over feeding behaviors, which has been hypothesized to indicate that low impulsivity, including low emotional impulsivity (urgency), may place certain individuals at risk for AN; however, this has not been fully genetically evaluated. We used genomic structural equation modeling and genome-wide association studies (GWASs) based on individuals of European ancestry (n = 72,517–903,147) to examine the latent genetic architecture between AN and several measures of impulsivity. Because AN is positively genetically associated with substance use disorders (SUDs), which are also strongly associated with impulsivity, we conditioned our analyses using GWAS data from four SUDs (alcohol, tobacco, cannabis, and opioid use disorders). AN was not significantly genetically correlated with impulsivity latent factors as indices of Barratt Impulsiveness Scale (BIS) or Urgency, Premeditation, Perseverance, Sensation Seeking, and Positive Urgency (UPPS) subscales (common impulsivity, *r_g_* = −0.07; urgency-specific impulsivity, *r_g_* = 0.14; and sensation seeking, *r_g_* = −0.07) but was significantly negatively genetically correlated with delay discounting (*r_g_* = −0.19) and lack of perseverance (*r_g_* = −0.15), even after controlling for SUDs (*r_g_* = −0.32 or *r_g_* = −0.25, respectively). This work suggests that delay discounting and lack of perseverance capture genetically informative dimensions of AN; clarifying shared etiologies could inform AN diagnosis and treatment mechanisms.

## Introduction

Anorexia nervosa (AN) is characterized by a fear of weight gain and a distorted body image, often accompanied by excessive self-control over restricted food intake and other weight loss-related behaviors (1). Up to 4% of female and 0.3% of male individuals are affected by AN, and the incidence among persons younger than 15 has increased in recent years (2). AN can cause serious adverse health outcomes, leading it to have the highest mortality rate of any psychiatric disorder, five times what is observed in the general population according to age and sex (3). While treatments for AN exist, their efficacy and overall recovery rates remain low (1). Elucidating risk factors contributing to AN development could illuminate the potential for novel treatment and prevention mechanisms.

Individuals with AN exhibit excessive control over feeding behavior for potential future reward (i.e., further weight loss), even when such behavior is life-threatening (4). Self-control is the opposite of impulsivity, which has been defined as thoughts or actions that are “poorly conceived, prematurely expressed, unduly risky or inappropriate to the situation, and that often result in undesirable consequences” (5). However, the construct of impulsivity is multifaceted (6). Impulsivity facets can be captured via self-reported questionnaires, such as the UPPS-P Impulsive Behavior Scale (7, 8) and Barratt Impulsiveness Scale (BIS-11) (9), and related constructs, such as delay discounting (DD), which is the tendency to favor smaller current rewards over larger future rewards and can be measured using a number of procedures (e.g (10–12)). Several studies have explored the association between AN and impulsivity/DD, often identifying *excessively low* levels of impulsivity/DD in patients with AN (phenotypic studies: e.g (13–18)), yet results continue to remain highly variable (15). A number of genetic studies have been conducted to evaluate this relationship as well (e.g (19–22)); however, the unique contribution of individual impulsivity facets and AN has not been explored. Therefore, the combination of high phenotypic variability and the limited number of genetic studies exploring this relationship prompts further investigation via novel genetic tools. Identifying overlapping genetic factors underpinning AN and specific impulsivity facets can offer novel insights into disease pathophysiology.

There is an extremely well-established relationship between impulsivity and propensity for various substance use disorders (SUDs) (19, 21, 23–25). The most recent study by Vilar-Ribó et al. demonstrated that both substance use and SUD traits showed distinct associations with different impulsivity facets (25). In turn, AN is also positively associated with SUDs (26–30). The systematic review by Bahji et al. reported a pooled lifetime and current prevalence of eating disorders with any comorbid SUDs of 21.9% (26). Furthermore, Mellentin et al. showed that SUDs lead to an additive effect on excess mortality in eating disorders (28). In order to begin to tease out these associations, we used multivariate statistical techniques and genetic data to further examine the relationship between AN, facets of impulsivity, DD, and SUDs.

## Methods

### Genome-wide association studies

All genome-wide association study (GWAS) summary statistics were based on individuals of European ancestry based on genetic similarity (31), as summarized below; a full list of sample sizes and sample types is included in **Supplementary Table S1**. Because GWASs map associations to a common reference panel (i.e., the human genome), this enables us to explore associations even when they were conducted in separate populations (with different ascertainment schemes).

#### Anorexia nervosa

We used summary statistics from the most recent independent GWASs of AN (32). The clinical sample included 16,992 cases and 55,525 controls of European ancestry. This sample came from the Psychiatric Genomics Consortium (PGC) Eating Disorders Working Group (https://pgc.unc.edu/for-researchers/download-results/).

#### Impulsivity

GWASs of impulsivity were based on a sample of up to 133,517 23andMe Inc. research participants (20, 21). These included measures from the UPPS-P Impulsive Behavior Scale (7, 8) and the BIS-11 scale (9). The 20-item brief version UPPS-P Impulsive Behavior Scale consists of five subscales (“lack of premeditation”, “lack of perseverance”, “positive urgency”, “negative urgency”, and “sensation seeking”). The 30-item BIS consists of three subscales (“attentional”, “motor”, and “non-planning”).

#### Delay discounting

We used summary statistics from a recent GWAS of DD from 23andMe (22). Although we did not have data about the frequency of AN among these 23andMe research participants, we presumed that it was low given the low population prevalence of this diagnosis. Higher scores indicate greater valuation of short-term versus long-term rewards, or “steeper” DD.

#### Substance use disorders

We used summary statistics from GWASs of cannabis use disorder (CUD) (33), tobacco use disorder (TUD) (34), opioid use disorder (OUD) (35), and problematic alcohol use (PAU) (36).

### Data analyses

**Figure 1:**
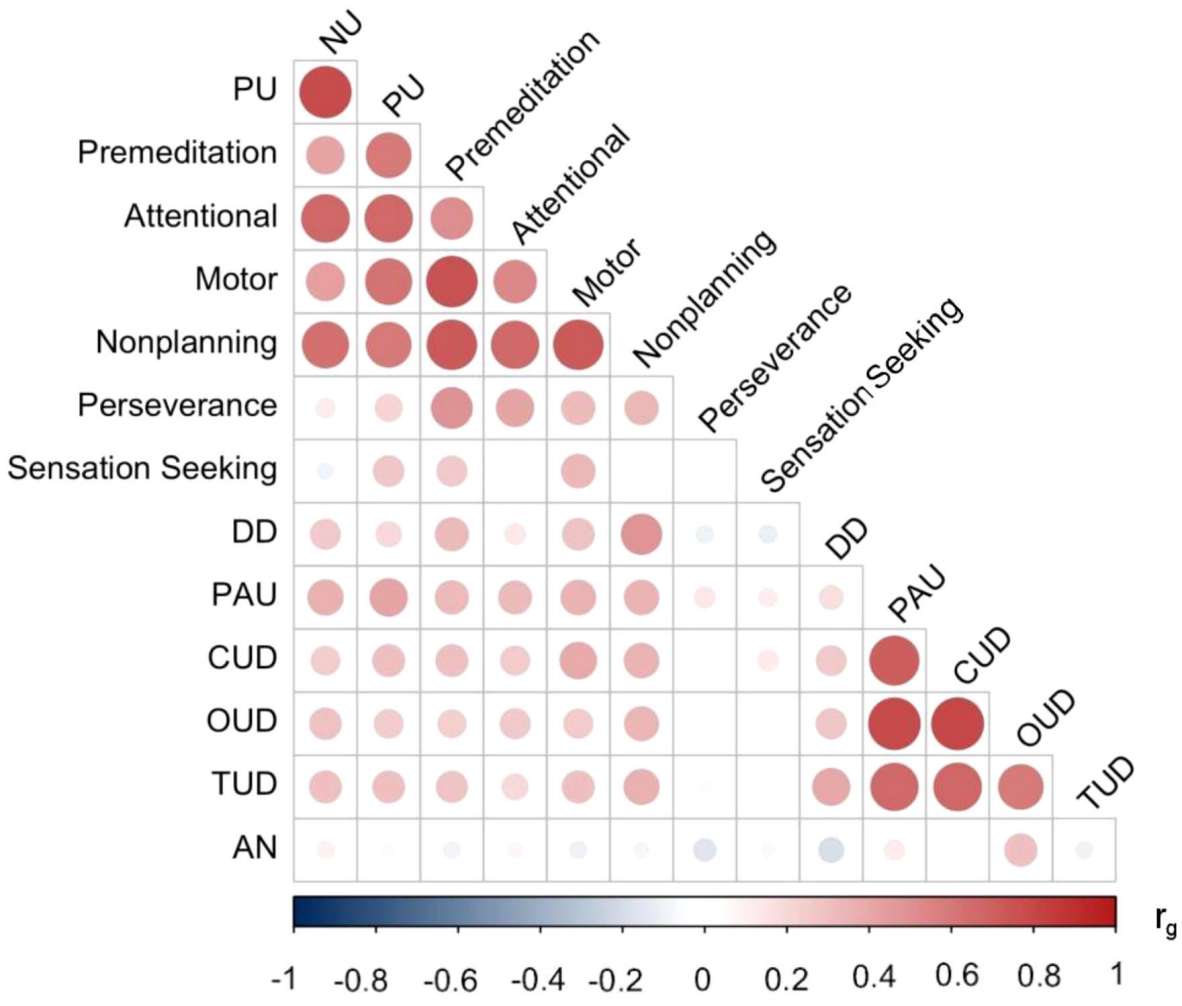
Genetic correlation (*r_g_*) matrix between all study variables. NU, UPPS-P Negative Urgency; PU, UPPS-P Positive Urgency; Premediation, BIS Premeditation; Attentional, BIS Attentional; Motor, BIS Motor; Nonplanning, BIS Nonplanning; Perseverance, BIS Lack of Perseverance; Sensation Seeking, BIS Sensation Seeking; DD, delay discounting; PAU, problematic alcohol use; CUD, cannabis use disorder; OUD, opioid use disorder; TUD, tobacco use disorder; AN, anorexia nervosa. The different-sized dots represent the magnitude of *r_g_* values. *r_g_* values and SE can be found in **Supplementary Table S2**.

We conducted all analyses in R version 4.1.1 (37). We used the genomic structural equation modeling (SEM) package (38), which applies SEM methods to GWAS summary statistics. Genomic SEM leverages linkage disequilibrium score regression (39) to generate a genetic correlation matrix between all traits for which summary statistics are available. **Figure 1** shows the genetic correlation matrix among all study variables. We modeled impulsivity and SUD factors based on our previous genomic SEM analyses (25, 40, 41). Although some models include perseverance and sensation seeking as impulsivity facets, our prior work showed that they are not strong contributors to a common impulsivity factor and are genetically distinguishable (40). Therefore, we modeled lack of perseverance and sensation seeking as separate indicators, along with DD (25, 40, 42).

Next, we fit SEMs to the data using genomic SEM, which drew on functionality from the *lavaan* R package (43). We fit two versions of this model: a) a correlated factor model where we estimated the genetic correlations between impulsivity, SUD, and AN factors, and b) a multiple regression, where AN was regressed on impulsivity and SUD factors. We used the default diagonally weighted least squares (DWLS) estimation method in these analyses. We determined the model fit based on chi-square tests (*χ* ^2^), the comparative fit index (CFI), the Akaike information criterion (AIC), and the standardized root mean square residual (SRMR). We expected good-fitting models to have CI > 0.95 (0.90 for acceptable fit), SRMR < 0.08, and smaller AIC values compared with competing nested models (Hu & Bentler, 1998). Good-fitting models also traditionally have non-significant *χ* ^2^ statistics. However, because sample sizes in GWASs are extremely large and *χ* ^2^ statistics are sensitive to sample size, we focused on other fit indices. We established the significance of individual parameter estimates with standard errors (SEs) and *p*-values.

### Data availability

GWAS summary statistics for AN and the SUD traits are publicly available. Data from 23andMe are available upon request (see https://research.23andme.com/dataset-access/). The R data files containing the genomic SEM matrices for all analyses are displayed at the following link: https://osf.io/4tjw5/. This allows for replication and analyses of competing models without obtaining the source data.

## Results

### Latent variable models of anorexia nervosa and impulsivity

**Figure 2:**
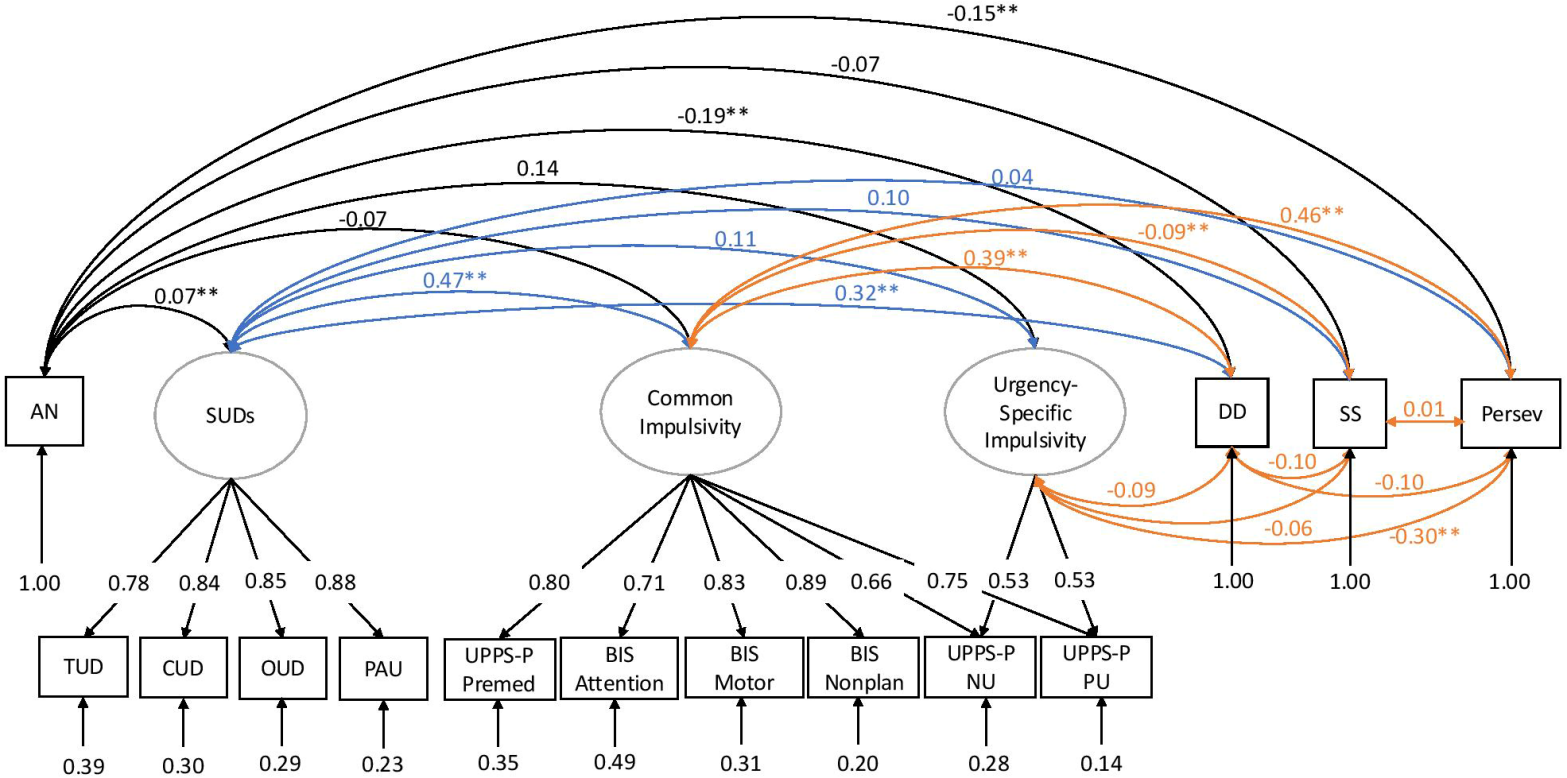
Genetic correlation model with AN, impulsivity facets, SUDs, delay discounting, sensation seeking, and lack of perseverance, adapted from prior studies (40). Ovals indicate latent factors, and squares indicate individual GWAS summary statistics. In this model, a “common impulsivity” factor successfully captured the shared variance across selected measures of the UPPS-P and BIS scales. To capture the particularly high correlation among UPPS-P negative urgency and UPPS-P positive urgency subscales, we included a second latent factor called “urgency-specific impulsivity”, which was fixed to be uncorrelated with genetic variance in common impulsivity. The four SUDs were modeled using a single factor (“substance use disorders”). The values under each trait represent the residual variances of the indicators. The colors are included for ease of visualization (e.g., black, correlations with AN; blue, correlations with SUDs; orange, correlations among impulsivity facets). UPPS-P NU, UPPS-P Negative Urgency; UPPS-P PU, UPPS-P Positive Urgency; UPPS-P Premed, UPPS-P Premediation; BIS Nonplan, BIS Nonplanning; SUDs, substance use disorders; PAU, problematic alcohol use; CUD, cannabis use disorder; OUD, opioid use disorder; TUD, tobacco use disorder; SS, BIS Sensation Seeking; Persev, BIS Lack of Perseverance; DD, delay discounting; AN, anorexia nervosa; GWASs, genome-wide association studies.

First, a correlational model of impulsivity facets, SUD, and AN was constructed. This model included two latent factors for impulsivity, capturing common variance across impulsive urgency and lack of premeditation (common impulsivity) and variance unique to impulsive urgency (urgency-specific impulsivity). DD, SS, and lack of perseverance were modeled as separate indicators based on our prior work showing that these constructs are genetically distinguishable (25, 40, 42). The four SUDs were modeled using a single factor (substance use disorders), similar to recent studies (41, 44). AN was included as a separate indicator. This model is displayed in **Figure 2** and had an acceptable fit (*χ* ^2^(x) = 1,035.32, *p* < 9.30E−178, CFI = 0.940, SRMR = 0.074).

AN was negatively genetically correlated with DD (*r_g_* = −0.19, SE = 0.039, *p* = 1.63E−06) and lack of perseverance (*r_g_* = −0.15, SE = 0.045, *p* = 7.51E−04) but not with the common impulsivity, urgency-specific factors, or SS. AN had a modest but significant positive genetic correlation with the SUD factor (*r_g_* = 0.07, SE = 0.027, *p* = 7.18E−03).

After conditioning on SUDs and impulsivity (by regressing AN on all other factors), AN was still negatively genetically correlated with delay discounting (*β* = −0.32, *p* = 4.63E−05) and lack of perseverance (*β* = −0.25, *p* = 5.52E−03). In contrast, we continued to observe non-significant negative genetic correlations between AN and the common and urgency-specific impulsivity factors (*β* = 0.14, *p* = 0.203, and *β* = 0.01, *p* = 0.99, respectively). In this model, SS was also significant with AN only after conditioning for SUDs (*β* = −0.14, *p* = 8.77E−03).

## Discussion

Using existing GWAS data, we investigated genetic associations between AN and multiple impulsivity facets. We found that AN was significantly negatively genetically associated with DD and lack of perseverance, while the genetic associations with common impulsivity, urgency-specific impulsivity, and SS were non-significant. These observations held even after controlling for the shared genetic variance among SUDs. This discovery, as we elaborate below, can have important implications for our understanding of the genetic susceptibility to AN, an illness with the highest mortality rate among all psychiatric disorders (3). It also illustrates that both extremes of impulsivity are associated with psychiatric disorders: steeper DD (i.e., greater discounting of delayed rewards) has been previously associated with SUDs and ADHD, among others (23, 45, 46), whereas the current report illustrates that shallower DD is associated with AN, and prior reports have similarly shown a relationship between low DD and obsessive compulsive disorder (OCD) (47). Likewise, the negative association between lack of perseverance and AN is countered by an increase in lack of perseverance found in borderline personality disorder (48).

Identifying common genetic relationships between AN and impulsivity facets can offer novel insights into disease pathophysiology. In addition to genetic correlations of individual traits (**Figure 1**), we also performed a multi-factorial analysis of impulsivity that included multiple facets via two well-established questionnaires, UPPS-P and BIS-11, and DD. We identified that AN was not significantly *genetically* associated with the UPPS/BIS measures of impulsivity, except lack of perseverance, which distinguished our results from previous *phenotypic* studies that identified both positive and negative associations between AN and attentional impulsivity, negative urgency, positive urgency, motor impulsivity, and sensation seeking (positive, e.g (14, 16, 17, 49–52); negative, e.g (13, 17, 53); neither, e.g (15)). In our study, SS was correlated with AN only after controlling for shared variance among SUDs. However, these findings support our previous work showing that emotional impulsivity, specifically urgency-specific genetic influences, is much more closely tied to internalizing traits than other psychiatric conditions, such as AN (40). There are a few possibilities for this discrepancy, one being ascertainment differences, namely, higher rates of mood disorders, as described in (17, 52), or that prior positive associations are indicative of consequences of the illness. Additionally, phenotypic associations are due to both genetic and environmental contributions. The lack of significant genetic associations observed here suggests that phenotypic associations observed by prior studies may reflect environmental rather than genetic factors.

In contrast, we found a negative genetic correlation with DD, in agreement with prior phenotypic studies (4, 54, 55). This association suggests that the increased capacity to delay reward could be an endophenotype for AN (4). Our findings also suggest that DD is a significant correlate of AN (56) since the datasets used for the genetic correlations were derived from independent cohorts (i.e., individuals from the impulsivity and DD datasets were not ascertained for AN). Notably, genomic SEM does not require that AN be directly represented in the impulsivity GWAS sample. Instead, it models the genetic covariance between traits using GWAS summary statistics, regardless of phenotypic co-occurrence within cohorts. Therefore, the observed associations reflect the shared genetic architecture, not sample overlap. However, the specific biological mechanisms underlying AN and DD remain unknown; multivariate approaches combining GWASs of AN and DD may help us identify specific genetic markers that could contribute to disease pathophysiology (57).

Studying the relationship between AN with DD and lack of perseverance could lead to novel insights into potential diagnostic mechanisms (15). First, DD could be used in combination with the existing AN diagnostic criterion (18, 54). Based on the lack of significant correlations (albeit with the same negative direction) with other impulsivity facets identified in this study, or increased/decreased associations with other impulsivity traits in prior phenotypic studies, AN may be best conceptualized as a mixture of behaviors of under-and-over control in the same individual, which should be considered when formulating diagnostic approaches (15). Intriguingly, high levels of DD and impulsivity facets are transdiagnostic traits for many psychiatric conditions, particularly those on the externalizing spectrum, such as SUDs (23) and ADHD (46).

Second, modulating DD could be considered for treatment approaches because of its role in both clinical presentation and outcomes (16, 17, 58). Prior studies have suggested that increasing DD has alleviated AN symptomatology (e.g., excessive focus on maintaining a low weight over time) (4, 15, 54, 59, 60). However, it is possible that the same is not true for other eating disorders, such as bulimia nervosa and binge-eating disorder, which possess a slightly different profile of associated impulsivity facets (16, 47, 55). Furthermore, the positive associations between DD/impulsivity and SUDs suggest that substance use should also be monitored when considering impulsivity facets as modifiable factors (47).

We observed a significant negative genetic association between AN and lack of perseverance. Another phenotypic study found that lack of perseverance was associated with restraint, eating concern, and shape concern when looking at associations of the facets of impulsivity and AN within a cohort of women (61). However, the extent to which this trait could serve as an endophenotype for AN is more of an unknown. Other studies have shown that lack of perseverance is a trait less related to emotions and more specifically characterizes patients with bulimia spectrum disorders more than AN, suggesting group differences (16, 49, 50). There is a lack of studies in the literature further phenotypically exploring the specific association with AN to draw more concrete conclusions about its clinical relevance.

The current literature describes that AN is typically characterized by low impulsivity and SUDs by high impulsivity, yet there is a positive correlation between AN and SUDs. The current study reinforces this by showing that the association between AN and SUDs persists after controlling for impulsivity. This relationship may be due to a number of factors, such as genetic risks, brain chemistry, family history, trauma, low self-esteem, depression, anxiety, and social pressures (62). However, previous phenotypic studies have reported this finding to be most prevalent in the binge eating/purging subtype of AN (26, 63). Further analysis of the different AN subtypes will further define the relationship between AN and SUDs and what populations are at the highest risk.

Our study is not without limitations. Differences between self-reported and behavioral measures of DD have been documented in AN patients (15), but our study only considered self-reported measures. In addition, there are two subtypes of AN, restrictive or binge/purge, with the binge/purge subtype showing a greater phenotypic association with impulsivity (17). However, we used summary statistics from an AN GWAS that was not subtype-specific. Additionally, there are other aspects of AN that we have not considered. Compulsivity is related to AN via behaviors of rumination thoughts toward starvation and rigidity in eating behavior (61, 64). Impulsivity and compulsivity may not be completely separable components (61); however, to date, there are no GWASs of compulsivity. Furthermore, GWASs were only conducted in individuals with European genetic similarity; therefore, it is unknown if our findings will generalize to other populations as larger non-European samples become available. Lastly, the associations examined here are based purely on genetic data, which may differ from those of an environmental nature. Longitudinal studies and more diversity in the GWASs of AN and its symptomatology could help identify sensitive periods where the role of impulsivity may be most salient in the prognosis of AN (56).

Our study has uncovered an overlapping genetic basis between AN and the impulsivity domains of DD and lack of perseverance. Based on the literature, a better understanding of the shared genetic and environmental etiologies between AN and these specific impulsivity facets could inform AN diagnostic and treatment strategies.

## Data Availability

The original contributions presented in the study are included in the article/**Supplementary Material**. Further inquiries can be directed to the corresponding author.
